# Hospitalization Utilization Metric After Major Oncologic Head and Neck Surgery With Microvascular Free Flap

**DOI:** 10.1002/ohn.70377

**Published:** 2026-08-17

**Authors:** Nana‐Hawwa Abdul‐Rahman, Anthony Tang, Zainab Balogun, Alexandria Harris, Shaum Sridharan, Matthew E. Spector, Carl H. Snyderman

**Affiliations:** ^1^ University of Pittsburgh School of Medicine Pittsburgh Pennsylvania USA; ^2^ Department of Otolaryngology University of Pittsburgh School of Medicine Pittsburgh Pennsylvania USA

**Keywords:** emergency department visit, head and neck cancer, hospital utilization, microvascular free flap reconstruction, quality of care, readmission

## Abstract

**Objective:**

The primary length of stay (PLOS) metric fails to capture added time, costs, and reduced patient quality of life (QOL) associated with readmissions. To address these limitations, we propose a novel metric, the hospitalization utilization metric (HUM), which integrates PLOS and readmission LOS (RLOS).

**Study Design:**

Retrospective review of 626 head and neck microvascular free flap (MVFF) cases from August 2019 to May 2024.

**Setting:**

University of Pittsburgh Medical Center.

**Methods:**

Main outcome measure was HUM, calculated as total length of stay (TLOS) (TLOS = [PLOS + RLOS]/60 days). High HUM, determined by median split, was >0.18.

**Results:**

Our 626‐patient cohort averaged 62.7 ± 11.9 years old. The majority of patients were male (68.2%), white (92.0%), with advanced tumors (III/IV: 74.1%), and public insurance (63.1%). The median (interquartile range [IQR]) PLOS was 9 (7‐14), median (IQR) HUM was 0.18 (0.13‐0.28), and the rate of 60‐day re‐presentation/readmission was 34.3%. Multivariable logistic regression identified postoperative complications (*β*: .08, *P* < .001; *β*: 4.44, *P* < .001, respectively) and private insurance (*β*: −.04, *P* < .02; −2.71, *P* < .01) as predictive factors associated with both higher HUM and PLOS (>9 days), respectively. Return to intensive care unit (ICU) (*β*: .08, *P* < .003) and duration of surgery (*β*: .005, *P* < .048) were independently associated with higher HUM, although both effect sizes are relatively small. Kaplan‐Meier survival curves revealed that higher HUM (*P* = .030) but not PLOS (*P* = .098) was associated with worse survival 1 year postoperatively.

**Conclusion:**

HUM provides a comprehensive assessment of healthcare utilization compared to PLOS and re‐presentation alone and may be a practical surrogate for patient QOL and healthcare costs.

Patients undergoing oncologic head and neck surgery have complex and highly specialized postoperative care needs that differ from other surgical specialties.^1^ These include airway management such as tracheotomy care, nutrition support, speech, and swallowing rehabilitation.^1^, ^2^ The complexity of postoperative care increases when microvascular free flap (MVFF) reconstruction is performed, given the added risk of both flap and donor site‐related complications. Collectively, these unique challenges place patients at high risk for unplanned hospital readmission after discharge.^3^ The reported readmission rate in the head and neck cancer literature ranges from 5% to 19.8%, with the highest rates observed in MVFF patients, where nearly one in five patients are readmitted.^4^, ^5^

Readmission is a widely recognized quality of care metric in healthcare delivery, as it reflects both patient outcomes and resource utilization. In recent years, healthcare systems and payers have prioritized strategies to reduce readmission,^6^ often through financial penalties or reimbursement models designed to incentivize higher‐quality and cost‐efficient care.^7^ Another commonly used quality of care metric is hospital length of stay (LOS).^8^, ^9^ Prolonged hospital stays have been linked to higher healthcare cost, high risk of hospital‐acquired complications, and a predictor of perioperative morbidity and readmission.^8^, ^9^, ^10^, ^11^, ^12^, ^13^, ^14^ Individually, both readmission and LOS capture important but distinct aspects of care quality. Readmission highlights complications occurring after discharge, while LOS reflects extended in‐hospital recovery immediately after surgery. While each measure is valuable in isolation, they both have limitations for fully assessing the total burden of hospitalization experienced by patients. Together, there may be benefits to combining the two metrics to create a more comprehensive measure of quality of care after surgery.

Accordingly, we propose a novel composite metric, the hospitalization utilization metric (HUM), which integrates both the primary length of stay (PLOS) and readmission LOS. We hypothesize that this composite measure will more accurately capture the cumulative burden of hospitalization, including its impact on healthcare costs, complication rates, and long‐term survival outcomes. Herein, the aim of this study is to develop a composite quality‐of‐care metric known as the HUM and evaluate its correlation with postoperative outcomes, including overall survival, healthcare costs, and postoperative complications in patients undergoing head and neck surgery with MVFF reconstruction.

## Methods

In this retrospective cohort study, we reviewed the clinical records of 626 patients who underwent oncologic head and neck surgery with MVFF reconstruction at our institution between August 2019 and May 2024. All eligible patients undergoing these procedures were included, and no specific procedure types were excluded, including recurrent or salvage cases.

A manual chart review of patients' electronic medical records was conducted. Initially, 638 patients were identified, and 12 patients were subsequently excluded due to missing data relevant to all analyses (primary or readmission LOS, follow‐up time). This study was approved by the University of Pittsburgh's Institutional Review Board (STUDY20050058). Variables of interest included patient demographics, tumor site (grouped into sinonasal/skull base vs oral cavity/oropharynx/larynx/hypopharynx/salivary gland), tumor stage (I/II vs III/IV), insurance type (public vs private), duration of surgery, estimated blood loss (mL), PLOS (days), 60‐day readmission rate, 60‐day emergency department (ED) visits, and tracheostomy status at discharge. PLOS was defined as the days from the date of surgery (DOS) to the date of discharge. Readmission LOS was defined as the total days spent in readmission within the first 60 days after DOS, and HUM was defined as the total number of days of hospitalization (PLOS, representation to ED, and readmission LOS) over 60 days. Readmissions were limited to events that occurred within 60 days after DOS that were related to the original surgery; unrelated conditions (such as myocardial infarction) or planned readmissions were not included. A 60‐day postoperative window was used because a standard 30‐day timeframe may be insufficient in this population. Patients undergoing complex head and neck oncologic surgery with MVFF reconstruction often experience prolonged initial hospital courses, and a substantial proportion of ED visits and readmissions occur between 30 and 60 days postoperatively. Therefore, extending the observation period to 60 days provides a more comprehensive assessment of clinically relevant postoperative outcomes. All included patients had follow‐up extending beyond the 60‐day postoperative period, allowing complete capture of outcomes within the study window.

Representations to the ED without readmission were included in HUM calculation as an additional day of hospitalization. Reasons for readmission within 60 days after DOS were classified as surgical reasons, medical reasons, or both. Surgical reasons involved complications directly related to the surgical site(s), including wound dehiscence, surgical site infection or abscess, flap or donor‐site concerns, postoperative bleeding, seroma or drainage issues requiring surgical evaluation. Medical reasons included non–wound‐related postoperative needs, such as gastrointestinal symptoms, feeding tube dysfunction, electrolyte abnormalities, and general postoperative supportive care needs. A high HUM (>0.18) and high PLOS (>9 days) cutoff was defined as a value greater than or equal to the median HUM or PLOS, respectively. Statistical analyses were conducted using R version 4.5.1. An alpha level of <.05 was considered statistically significant. Descriptive statistics were used to summarize the demographics of the patient population. Chi‐square tests and Fisher exact tests were used to analyze categorical variables. Univariable analysis was done using chi‐square and *t* tests. Multivariable logistic and linear regression was performed, allowing into the model variables that were associated with either re‐presentation/readmission, increased PLOS, or increased HUM at the <0.1 significance level in univariable analysis. This strategy was chosen a priori to maintain consistency across models and to avoid omitting clinically relevant variables across these three related outcomes. Survival analysis excluded 14 patients that died <60 days after surgery, and an additional landmark analysis was completed using day 60 as time point 0 to account for time biases. Survival analysis was conducted using the Kaplan‐Meier method, and survival curves were compared using the log‐rank test.

## Results

### Demographics

Our 626‐patient cohort averaged 62.7 ± 11.9 years old. The majority were male (68.2%), white (92.0%), with advanced tumors (III/IV: 74.1%), and on public insurance (63.1%). The median (interquartile range [IQR]) PLOS was 9 (7‐14), median (IQR) HUM was 0.18 (0.13‐0.28), and rates of 60‐day re‐presentation/readmission were 34.3%. Reasons for readmission include surgical reasons (32.8%), medical reasons (57.8%), and both (9.3%) (**Tables** 1 and 2).

**Table 1:** Demographics and Patient Presentation Table

	N = 626
Variables	Mean (SD)
Age, y	62.7 (11.9)
BMI	27.1 (12.5)

	N (%)
Sex	
Male	427 (68.2)
Female	199 (31.8)
Race	
White	576 (92.0)
Black	19 (3.0)
Asian	7 (1.2)
Other/unknown	24 (3.8)
T stage	
0/1/2	162 (25.9)
3/4	464 (74.1)
Site	
Oral cavity/oropharynx/larynx/hypopharynx/salivary gland	608 (97.1)
Sinonasal/skull base	18 (2.9)
Insurance type	
Public	395 (63.1)
Private	201 (32.1)
Unknown	30 (4.8)

Abbreviations: BMI, body mass index; SD, standard deviation.

**Table 2:** Clinical Characteristics Table

	N = 626
Variables	Mean (SD)
Duration of surgery, h	10.1 (2.7)
EBL, mL	239 (321)

	Median (IQR)
Hospitalization utilization metric	0.18 (0.13‐0.28)
Primary LOS, d	9 (7‐14)

	N **(%)**
Readmission/representation to ED	
None	422 (67.4)
Yes	204 (32.6)
Reasons for readmission/representation to ED	
Surgical reason	67 (32.8)
Medical reason	118 (57.8)
Both	19 (9.3)
Any complication	
No	365 (58.3)
Yes	261 (41.7)
Tube feeds	
No	161 (25.7)
Yes	465 (74.3)
Tracheostomy tube at discharge	
No	397 (63.4)
Yes	229 (36.6)

Abbreviations: EBL, estimated blood loss; ED, emergency department; IQR, interquartile range; LOS, length of stay; SD, standard deviation.

### Associations of Predictive Factors With Readmission, PLOS, and HUM

#### Univariate Analyses

See **Table** 3 for the results of the univariate analyses. Higher tumor stage (*P* < .02), longer surgeries (*P* < .004), public insurance (*P* < .003), tracheostomy at discharge (*P* < .04), intensive care unit (ICU) admission (*P* < .01), and any postoperative complications (*P* < .001) were associated with a higher HUM. All the aforementioned variables were also significant for PLOS. None of the variables were associated with readmission/ED visits.

**Table 3:** Univariate Analysis^a^

	Readmission/representation to ED			HUM			Primary LOS		
Variables	No (mean [SD])	Yes (mean [SD])	*P*‐value	N	Corr	*P*‐value	N	Corr	*P*‐value
Age	63.0 (11.2)	62.1 (13.0)	.42	626	0.06	.12	625	0.05	.25
BMI	27.2 (14.0)	27.0 (9.2)	.78	626	0.08	**.05**	625	0.09	**.03**
Duration of surgery, h	9.9 (2.8)	10.3 (2.5)	.10	626	0.11	**.004**	625	0.10	**.01**
EBL	224.2 (147)	265.2 (509)	.26	625	0.00	.97	624	−0.01	.73
ADI national	69.5 (21.3)	69.4 (22.7)	.99	582	0.05	.28	581	0.05	.21
ADI state	6.5 (2.6)	6.5 (2.8)	.92	582	0.06	.15	581	0.06	.13

	No (n [%])	Yes (n [%])	*P*‐value	N	Mean (SD)	*P*‐value	N	Mean (SD)	*P*‐value
T stage			.17			**.02**			**<.001**
0/1/2	114 (27.7)	48 (22.3)		162	0.21 (0.19)		162	10.7 (8.2)	
3/4	297 (72.3)	167 (77.7)		464	0.25 (0.17)		463	13.0 (9.4)	
Gender			.70			.84			.70
Male	283 (68.9)	144 (67.0)		427	0.24 (0.18)		426	12.5 (9.3)	
Female	128 (31.1)	71 (33.0)		199	0.23 (0.17)		199	12.2 (8.9)	
Insurance			.40			**.003**			**<.001**
Private	138 (35.0)	63 (31.2)		201	0.21 (0.14)		201	10.7 (6.4)	
Public	256 (65.0)	139 (68.8)		395	0.25 (0.18)		394	13.1 (10.0)	
Race			.52			.74			.48
White	377 (95.2)	199 (96.6)		576	0.23 (0.17)		575	12.2 (8.9)	
Black	13 (3.3)	6 (2.9)		19	0.23 (0.21)		19	11.9 (8.9)	
Asian	6 (1.5)	1 (0.5)		7	0.28 (0.21)		7	16.3 (12.9)	
Cancer site			.88			.96			.28
Oral cavity, oropharynx, larynx, hypopharynx, salivary	400 (97.3)	208 (96.7)		608	0.24 (0.18)		607	12.4 (9.2)	
Sinonasal/skull base	11 (2.7)	7 (3.3)		18	0.24 (0.17)		18	10.2 (8.3)	
Tube feeds at discharge			.26			.50			.56
No	112 (27.3)	49 (22.8)		161	0.23 (0.21)		161	12.0 (10.9)	
Yes	299 (72.7)	166 (77.2)		465	0.24 (0.16)		464	12.5 (8.5)	
Tracheostomy tube at discharge			.37			**.04**			**.03**
No	255 (62.0)	142 (66.1)		397	0.22 (0.16)		397	11.7 (8.8)	
Yes	156 (38.0)	73 (33.9)		229	0.26 (0.21)		228	13.5 (9.6)	
Required return to ICU from floor			.36			**.01**			**.01**
No	367 (93.6)	187 (91.2)		554	0.22 (0.17)		553	11.9 (8.9)	
Yes	25 (6.4)	18 (8.8)		43	0.33 (0.25)		43	16.1 (10.0)	
Any complication			.51			**<.001**			**<.001**
No	244 (59.4)	121 (56.3)		365	0.19 (0.11)		365	10.0 (6.2)	
Yes	167 (40.6)	94 (43.7)		261	0.30 (0.23)		260	15.7 (11.4)	

Bold values indicate *P* < .05.

Abbreviations: ADI, area deprivation index; BMI, body mass index; EBL, estimated blood loss; ED, emergency department; HUM, hospitalization utilization metric; ICU, intensive care unit; LOS, length of stay.

^a^ Univariable statistical tests used include independent sample *t* tests, chi‐square, one‐way analysis of variance (ANOVA), and correlation tests.

#### Multivariable Analyses

On multivariable logistical regression after controlling for body mass index (BMI), insurance, tumor stage, cancer site, ICU admission, duration of surgery, tracheostomy at discharge, and postoperative complications, only postoperative complications (*β*: .08, *P* < .001; *β*: 4.44, *P* < .001, respectively), and private insurance (*β*: −.04, *P* < .02; −2.71, *P* < .01) were associated with a higher HUM and PLOS, respectively. Return to ICU (*β*: .08, *P* < .003) and duration of surgery (*β*: .005, *P* < .048) were independently associated with a high HUM; however, both associations had small effect sizes (**Table** 4).

**Table 4:** Multivariable Analysis^a^

	Readmission/representation to ED			HUM			Primary LOS		
Variables	OR	95% CI	*P*‐value	Beta	*t*	*P*‐value	Beta	*t*	*P*‐value
BMI	1.00	0.98‐1.01	.859	0.001	1.738	.0827	0.052	1.911	.056
Duration of surgery, h	1.07	1.00‐1.14	.052	0.005	1.984	**.0478**	0.198	1.477	.140
T stage (3/4)	1.32	0.87‐2.00	.197	0.028	1.828	.0680	1.494	1.819	.069
Insurance (private)	0.87	0.59‐1.26	.451	−0.043	−3.106	**.0020**	−2.699	−3.612	**<.001**
Cancer site (vs sinonasal/skull base)	1.18	0.42‐3.33	.755	0.008	0.212	.8326	−1.899	−0.892	.373
Trach at discharge (yes)	0.78	0.54‐1.14	.206	0.002	0.135	.8927	0.345	0.456	.649
Return to ICU (yes)	1.43	0.73‐2.81	.299	0.078	3.043	**.0025**	2.378	1.693	.091
Any complication	0.98	0.67‐1.43	.916	0.084	6.015	**<.001**	4.436	5.846	**<.001**

Bold values indicate *P* < .05.

Abbreviations: BMI, body mass index; ED, emergency department; HUM, hospitalization utilization metric; ICU, intensive care unit; LOS, length of stay; OR, odds ratio.

^a^ Includes variables with *P*‐value < .1 from univariate analysis for either readmission/representation to ED, HUM, or primary LOS.﻿﻿

### Survival Analysis

**Figures** 1, 2, 3 show the 1‐year Kaplan‐Meier curves for HUM, PLOS, and readmission/ED visits, excluding patients who died within 60 days and day 0 starting at postoperative day 60. For HUM, 1‐year survival was 89.6% (95% CI: 86.0‐93.4) for low HUM patients and 83.3% (95% CI: 78.8‐88.0) for high HUM patients (*P* = .03). For PLOS, 1‐year survival was 88.8% (95% CI: 85.0‐92.7) for low PLOS patients and 84.1% (95% CI: 79.7‐88.7) for high PLOS patients (*P* = .098). For readmission/ED visits, 1‐year survival was 86.7% (95% CI: 83.1‐90.4) for patients with no readmission/ED visit within 60 days and 86.2% (95% CI: 81.3‐91.4) for patients with readmission/ED visit within 60 days (*P* = .86).

**Figure 1:**
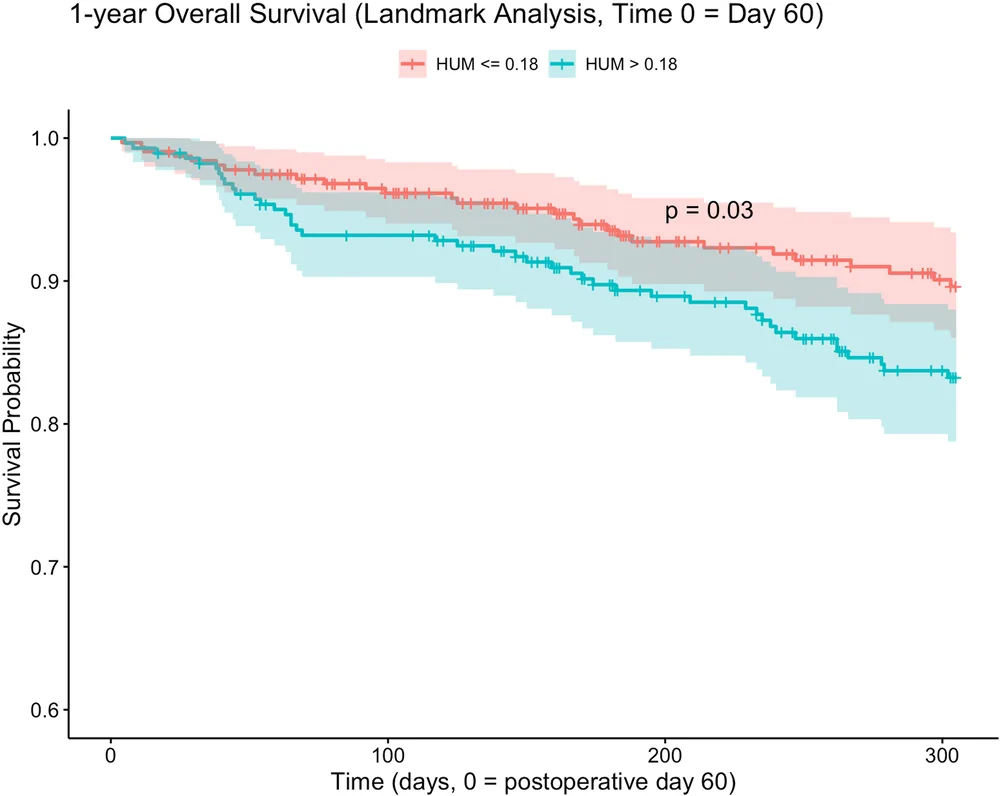
Kaplan‐Meier survival curve, 1‐year overall survival for high hospitalization utilization metric (HUM).

**Figure 2:**
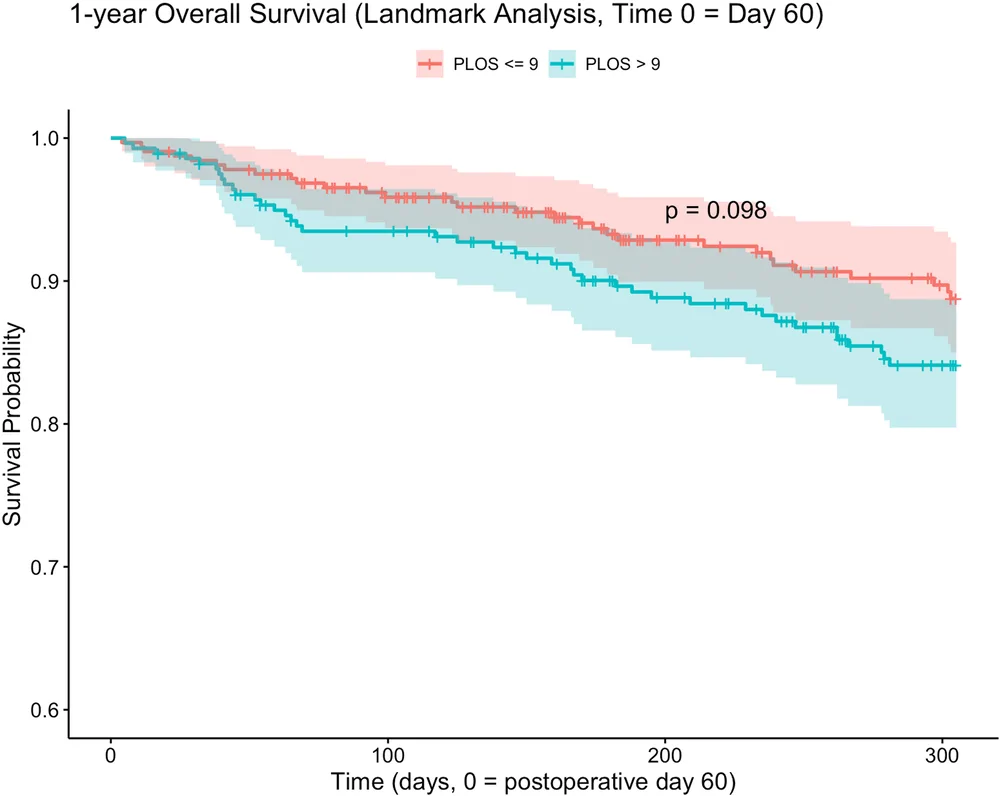
Kaplan‐Meier survival curve, 1‐year overall survival high primary length of stay (PLOS).

**Figure 3:**
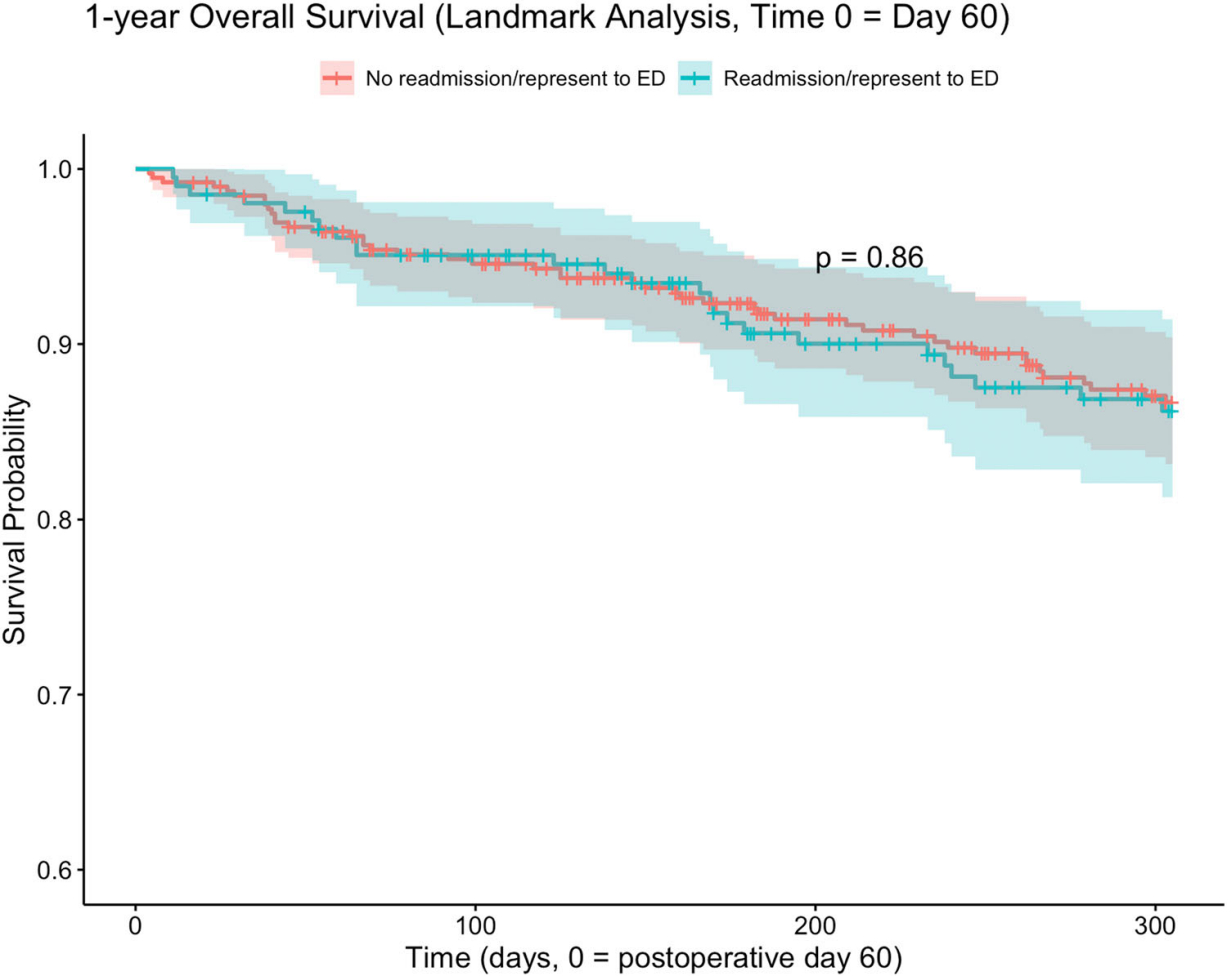
Kaplan‐Meier survival curve, 1‐year overall survival readmission/represent. ED, emergency department.

## Discussion

In this single‐institution cohort of 626 patients undergoing oncologic head and neck surgery with MVFF reconstruction, we found that HUM, which integrates PLOS and readmission LOS, was strongly associated with postoperative complications, insurance status, and 1‐year survival. Interestingly, only HUM was significantly associated with overall 1‐year survival. An additional landmark analysis confirmed that time bias did not influence the survival analysis, as HUM remained significantly associated with 1‐year survival (*P* = .03) while PLOS and readmission remained non‐significant when postoperative day 60 was used as time zero. Our findings are consistent with reports in other surgical specialties where composite hospitalization measures have similarly improved prediction of patient‐centered outcomes. For example, Tang et al demonstrated that the 30‐day HUM was a noninferior metric to PLOS and readmission, with the potential to provide a more complete quantification of outcomes and better capture the impact of hospital interventions in patients undergoing pituitary surgery.^15^ The authors posited that HUM could integrate both the initial hospitalization and any readmissions, thus reflecting the total hospital resource utilization and patient experience more accurately than LOS or readmission alone. They concluded that HUM may be preferable for evaluating interventions and outcomes in surgical populations, though further validation is needed across other specialties.^15^ Furthermore, Li et al's study highlighted the limitations of using LOS or readmission rates in isolation, noting that these metrics do not fully capture the complexity of patient trajectories, especially as reductions in LOS may shift complications to the post‐hospitalization period.^16^ Additionally, several studies have revealed the inherent limitations of LOS and readmission as standalone measures, observing that much of the variation in LOS is not explained by clinical factors and that readmission rates may not reliably reflect hospital quality.^17^, ^18^, ^19^, ^20^ Interestingly, Lingsma et al's study proposed a composite measure that combined LOS, readmission, and mortality to provide a more reliable and comprehensive view of hospital performance.^21^ Collectively, these studies underscore the growing recognition that composite metrics such as HUM may more faithfully represent postoperative risk and healthcare resource use.

Although HUM and TLOS are closely correlated, HUM provides a time‐normalized measure that captures the intensity of hospital use over a fixed 60‐day window. By contrast, PLOS reflects only the initial hospitalization and fails to account for the additive burden of readmission, and a simple readmission (yes/no) metric ignores duration entirely. As one of the first studies to establish the superiority of HUM over PLOS, we recognize that this work represents an early step in defining HUM's role in postoperative care. Nevertheless, our data suggest that HUM is a pragmatic tool for risk stratification such that patients with high HUM more often had advanced disease, postoperative complications, public insurance, or ICU stays. Incorporating HUM into perioperative planning could therefore help identify high‐risk individuals and guide targeted interventions aimed at reducing complications, readmissions, and overall hospital utilization. Additional investigation is needed to fully understand the association between HUM and 1‐year survival.

### Strengths

This study has several notable strengths. It represents one of the largest single‐institution cohorts of patients undergoing oncologic head and neck surgery with MVFF reconstruction, with detailed clinical and survival data collected over a 60‐day postoperative period. The consistent surgical team and standardized institutional care pathways reduce heterogeneity, and the use of multivariable modeling strengthens the internal validity of our findings. Importantly, this is among the first investigations in otolaryngology to propose and validate a composite HUM, providing an innovative, time‐normalized metric that integrates PLOS and readmission LOS to better reflect the total burden of care.

Our findings suggest that HUM is a practical, time‐normalized metric that captures the cumulative burden of hospitalization more comprehensively than PLOS or readmission alone. Looking ahead, several opportunities exist to expand and validate this work. First, multicenter and prospective studies across diverse healthcare systems are needed to confirm the reproducibility and external validity of HUM and to explore its performance within the field of otolaryngology and other surgical subspecialties. Second, future research should evaluate how HUM can be incorporated into risk prediction models and clinical workflows such as discharge planning algorithms, bundled payment structures, or value‐based reimbursement programs to further guide targeted interventions for high‐risk patients. Additional studies might also test alternative time windows (eg, 30 or 90 days) or dynamic HUM calculations that update in real time to support perioperative decision‐making.

### Limitations

A few limitations should be acknowledged. First, the retrospective single‐center design may limit generalizability, as referral patterns, discharge practices, and payer mixes vary across institutions. Second, our reliance on electronic medical records may introduce information bias, and unmeasured confounding variables cannot be fully excluded. Specifically, readmissions were limited to those captured within our healthcare system's electronic medical records. Third, quality of life was inferred from resource utilization rather than measured with patient‐reported outcome measures, and long‐term survival beyond 1 year was not assessed. Fourth, further validation across multiple centers, different surgical populations, and alternative timeframes is needed to confirm its robustness and external applicability. Lastly, variables with small effect sizes, such as postoperative complications, return to ICU, private insurance, and duration of surgery, demonstrated statistically significant associations but may not be clinically relevant. Despite these constraints, the large sample size, comprehensive capture of postoperative events, and consistent analytic approach provide confidence that HUM is a reliable and clinically meaningful metric for assessing quality and efficiency of care after complex head and neck surgery.

## Conclusion

In conclusion, this single‐institution study demonstrates that HUM independently predicts cost of care, postoperative complications, and 1‐year survival. HUM reflects the total hospitalization burden for patients undergoing oncologic head and neck surgery with MVFF reconstruction. In survival analysis, high HUM, but not PLOS, was significantly associated with worse overall 1‐year survival after surgery. By combining the duration of initial hospitalization and readmissions within a fixed 60‐day window, HUM offers a simple yet comprehensive metric that can enhance quality assessment, inform policy, and drive targeted strategies to improve patient outcomes and reduce healthcare costs. These findings lay the building blocks for broader, stronger adoption and validation of HUM as an impactful indicator of surgical care quality and efficiency.

## Author Contributions

**Nana‐Hawwa Abdul‐Rahman**, conceptualization and design, data curation, investigation, methodology, validation, writing and editing of manuscript; **Anthony Tang**, statistical analysis, writing and editing of manuscript; **Zainab Balogun**, writing and editing of manuscript; **Alexandria Harris**, data curation. **Shaum Sridharan**, writing and editing of manuscript; **Matthew E. Spector**, validation, data curation, writing and editing of manuscript; **Carl H. Snyderman**, conceptualization and design, methodology, supervision, validation, writing and editing of manuscript.

## Disclosures

### Competing interests

The authors declare no conflicts of interest.

### Funding source

None.
